# Diagnosis and management of non-IgE-mediated cow’s milk allergy in infancy - a UK primary care practical guide

**DOI:** 10.1186/2045-7022-3-23

**Published:** 2013-07-08

**Authors:** Carina Venter, Trevor Brown, Neil Shah, Joanne Walsh, Adam T Fox

**Affiliations:** 1 The David Hide Asthma and Allergy Research Centre, Newport, Isle of Wight, UK; 2 School of Health Sciences and Social Work, University of Portsmouth, Portsmouth, UK; 3 The Children’s Allergy Service, The Ulster Hospital, Ulster, Northern Ireland, UK; 4 Department of Gastroenterology, Great Ormond Street Hospital for Children NHS Foundation Trust, University College London, London, UK; 5 KU Leuven, Translational Research Centre for Gastrointestinal Disease (TARGID), Leuven, Belgium; 6 Castle Partnership, Gurney Surgery, Norwich, UK; 7 MRC & Asthma UK Centre in Allergic Mechanisms of Asthma, King’s College London, London, UK; 8 Department of Paediatric Allergy, Guy’s and St Thomas’ Hospitals NHS Foundation Trust, London, UK

**Keywords:** Cow’s milk allergy, Primary care, Food allergy, Diagnosis, Management, Hypoallergenic formula

## Abstract

The UK NICE guideline on the Diagnosis and Assessment of Food Allergy in Children and Young People was published in 2011, highlighting the important role of primary care physicians, dietitians, nurses and other community based health care professionals in the diagnosis and assessment of IgE and non-IgE-mediated food allergies in children. The guideline suggests that those with suspected IgE-mediated disease and those suspected to suffer from severe non-IgE-mediated disease are referred on to secondary or tertiary level care. What is evident from this guideline is that the responsibility for the diagnostic food challenge, ongoing management and determining of tolerance to cow’s milk in children with less severe non-IgE-mediated food allergies is ultimately that of the primary care/community based health care staff, but this discussion fell outside of the current NICE guideline. Some clinical members of the guideline development group (CV, JW, ATF, TB) therefore felt that there was a particular need to extend this into a more practical guideline for cow’s milk allergy. This subset of the guideline development group with the additional expertise of a paediatric gastroenterologist (NS) therefore aimed to produce a UK Primary Care Guideline for the initial clinical recognition of all forms of cow’s milk allergy and the ongoing management of those with non-severe non-IgE-mediated cow’s milk allergy in the form of algorithms. These algorithms will be discussed in this review paper, drawing on guidance primarily from the UK NICE guideline, but also from the DRACMA guidelines, ESPGHAN guidelines, Australian guidelines and the US NIAID guidelines.

## Introduction

In 2007 the World Health Organisation (WHO) formally acknowledged that allergy has become the No. 1 environmental epidemic disease facing children of the developed world [1]. A review paper by the World Allergy Organization [2] estimated that 1.9% to 4.9% of children suffer from cow's milk protein allergy (CMA), yet perceived food allergy could be up to 10 times higher than that confirmed by appropriate tests [3,4].

Infants are exposed to cow’s milk protein via the maternal diet if breast-fed, via standard infant formula, or when solids are introduced. It is, therefore, not surprising that cow’s milk is often identified as a possible cause for skin and gut problems, particularly in early infancy [3].

Recent evidence indicates that the diagnosis and management of CMA in UK primary care, has room for significant improvement. During 2009, 1000 infants diagnosed as CMA were randomly selected from the UK GP Health Improvement Network (THIN) database and subsequently several papers have been published analysing the clinical management of these infants in primary care. Significant under diagnosis, delayed diagnosis and incorrect diagnosis were clearly demonstrated. Also, the initial choice of replacement formula and the decision to refer on or not, showed worrying inconsistencies [5].

**Figure 1:**
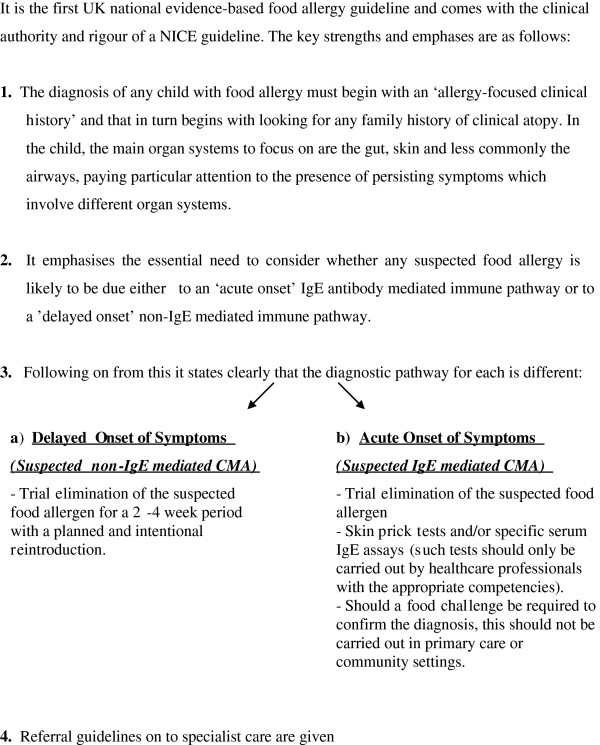
Strengths and main clinical emphases of the NICE guideline.

This work, along with other similar evidence, caused the UK Department of Health to commission a National Institute for Health and Clinical Excellence (NICE), Guideline on Food Allergy with a very specific and targeted scope, clearly described in its full title, ‘Diagnosis and assessment of food allergy in children and young people in primary care and community settings [6].’ This guideline [6], produced in 2011, recommends that many manifestations of food allergy could be managed in primary care. However, this necessitates primary care physicians (GPs in the UK), dietitians, nurses and other community based health care professionals having correct and up-to-date information on the different clinical presentations of CMA in order to make an accurate diagnosis, establish a management plan and ensure onward referral when indicated (Figure 1).

## Review

### Methodology

The NICE guideline was written to direct the diagnosis of all food allergies. CMA is however, the most clinically complex individual food allergy and therefore causes significant challenges in both recognising the many differing clinical presentations and also the varying approaches to management, both at primary care and specialist level. A subgroup of the clinicians on the NICE guideline development group (CV, JW, ATF, TB) felt that there was therefore a particular need to extend this into a more practical guideline for cow’s milk allergy for UK Primary Care use. This need was further emphasized by the publication of international and European guidelines on cow’s milk allergy [2,7-9]. This subgroup, with the additional expertise of a paediatric gastro-enterologist (NS) has produced a UK Primary Care Guideline in the form of practical algorithms.

Prior to the development of this Primary Care Guideline, the group discussed important questions that they wanted to address and which clear, practical messages they wanted to convey to UK primary care. These were:

How to distinguish between:

1) IgE-mediated and non-IgE-mediated presentations of CMA.

2) Severe and mild to moderate clinical expressions of CMA.

To provide guidance on formula choice in the initial diagnosis of CMA based on the current international guidelines.

Give guidance about the ongoing management of mild to moderate non-IgE-mediated CMA in primary care.

A literature search was conducted to ensure that all major food allergies and cow’s milk allergy guidelines published in the past five years were included. These included the World Allergy Organisation’s Guidelines on Cow’s Milk Allergy [2], the NIAID Food Allergy Guidelines from the US [10], the UK NICE Guideline on Food Allergy in Children and Young People [6], the ESPGHAN guidelines on the diagnosis and management of cow’s milk allergy [7]and the Australian consensus statement on the diagnosis and management of cow’s milk allergy [11]. All these papers were informed by extensive systematic reviews of the literature and the group (CV, TB, JW, NS, ATF), felt that they were rigorous enough to build this proposed additional practical guideline on. It is intended to complement the NICE Food Allergy Guideline.

### Prevalence of CMA

Population based studies report that the prevalence of Cow’s Milk Allergy (CMA) ranges from 1.9 – 4.9% in young children [2]. UK data from 2008 indicated 2.3% of 1–3 year olds suffer from CMA, the majority of these presenting with non-IgE-mediated CMA [3]. A meta-analysis by Rona et al. [4] reported that Cow’s milk (CM) is one of the most common foods which is responsible for allergic reactions in European children. In general, the prognosis for CMA is good, with up to 80-90% of children developing tolerance before three years of age [12]. However, CMA may persist up to school age and may be associated with the later development of other allergic diseases such as asthma, rhinoconjunctivitis, and atopic dermatitis [13], as well as other disease manifestations such as recurrent abdominal pain [14]. It is also well-known that perceived prevalence may be much higher [4,12] than that confirmed by appropriate tests. Cow’s milk formula or cow’s milk containing foods play an important role in the nutritional intake of children particularly in early infancy. Onset after infancy has also been uncommonly reported [3].

### Nomenclature

The first step in making the correct diagnosis and managing infants and children with cow’s milk allergy is to have a good understanding of the immune mechanisms involved. According to the European Academy for Allergy and Clinical Immunology (EAACI) and the World Allergy Organisation (WAO) [15], a hypersensitivity reaction to cow’s milk can be referred to as cow’s milk allergy if it involves the immune system. Non-allergic cow’s milk hypersensitivity (lactose intolerance) on the other hand, does not involve the immune system. Cow’s milk allergy is further divided into IgE-mediated cow’s milk allergy and non-IgE-mediated cow’s milk allergy [7]. There is however clinical overlap between some presentations of cow’s milk allergy as indicated by the US food allergy guidelines [10].

### The different manifestations of CMA

According to the UK NICE guideline [6], food allergy can manifest as a number of different clinical presentations, mainly affecting the skin, gastro-intestinal tract and respiratory systems.

The NICE guideline [6] emphasises that food allergies should be particularly considered 1) in infants where there is a family history of allergic disease (but the absence of a family history of allergy does not exclude the possibility of becoming allergic), 2) in infants where symptoms are persistent and affecting different organ systems and 3) in infants who have been treated for moderate to severe atopic eczema, gastro-oesophageal reflux disease (GORD) or other persisting gastrointestinal symptoms (including ‘colic’ , loose stools, constipation), but have not responded to the usual initial therapeutic interventions.

**Figure 2:**
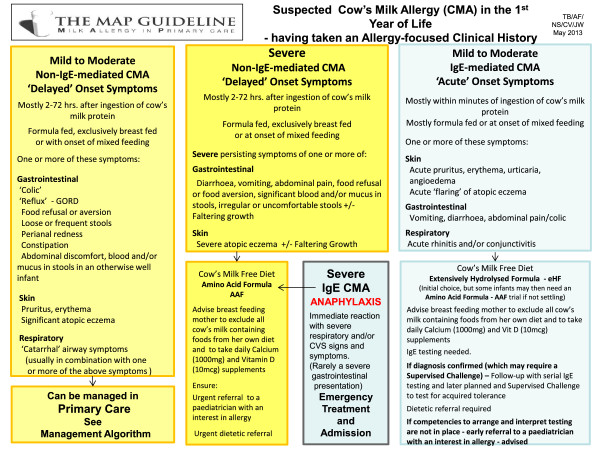
Different presentations of cow’s milk allergy in infancy.

In Figure 2 of the algorithms, we have divided IgE and non-IgE-mediated CMA into “mild-moderate presentations” and “severe presentations” to aid in the diagnostic process, management of CMA and appropriate onward referral. Therefore, most importantly, Figure 2 gives a clear message about which infants can be safely diagnosed and managed in UK primary care without any onward referral to secondary or tertiary care.

### Diagnosis of Cow’s milk allergy

#### History taking

Taking an allergy focused history forms the cornerstone of the diagnosis of food allergies including CMA and the UK NICE guideline [6] recommends that questions should be asked regarding:

•Any family history of atopic disease in parents or siblings.

•Any personal history of early atopic disease.

•The infant's feeding history.

•Presenting symptoms and signs that may be indicating possible CMA.

•Details of previous management, including any medication and the perceived response to any management.

•Was there any attempt to change the diet and what was the outcome?

An EAACI task force also dealt with the important questions that should be asked during an allergy focused diet history, and will be available later this year.

Following on from these questions is the important step to attempt to differentiate between possible IgE and non-IgE -mediated allergies (Figure 2) and which “tests” to do.

#### IgE-Mediated CMA

**Figure 3:**
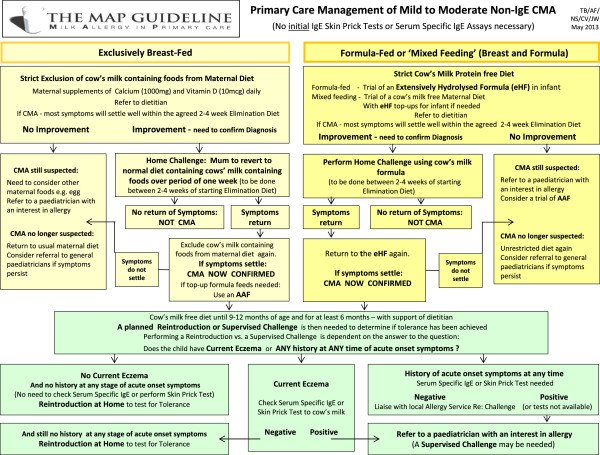
Diagnosis and management of mild to moderate non-IgE CMA in UK primary care.

For the diagnosis of IgE-mediated CMA, the use of skin prick tests (SPT) or specific serum IgE tests are recommended, but these should only be performed by those competent to interpret the tests [16]. It is important to understand that a positive SPT or specific serum IgE test merely indicates sensitisation and does not confirm clinical allergy. However, a positive test coupled with a clear history of a reaction should usually be sufficient to confirm a diagnosis. Although a diagnostic oral food challenge (after a short period of cow’s milk avoidance) may not be required in most of these cases, if such a challenge is conducted, it may need to be performed in a supervised setting in the majority of cases. Liasion with or referral to a local paediatric allergy team is recommended (see Figure 3).

#### Non-IgE -Mediated CMA

There are no validated tests for the diagnosis of non-IgE CMA, apart from the planned avoidance of cow’s milk and cow’s milk containing foods, followed by reintroduction as a home challenge to confirm the diagnosis [17]. Home reintroduction/challenges may not be acceptable in children with severe forms of non-IgE- mediated cow’s milk allergy, and these children should be referred to secondary/tertiary care [6].

#### The role of dietary interventions in the diagnosis of IgE and non-IgE-mediated CMA

Maternal avoidance of cow’s milk in the case of breast fed infants, or choosing an appropriate formula for bottle fed/partially bottle fed infants are crucial steps in the diagnosis of CMA. Mothers excluding cow's milk from their diet should be supplemented with calcium and vitamin D [18] (Figure 2).

**Figure 4:**
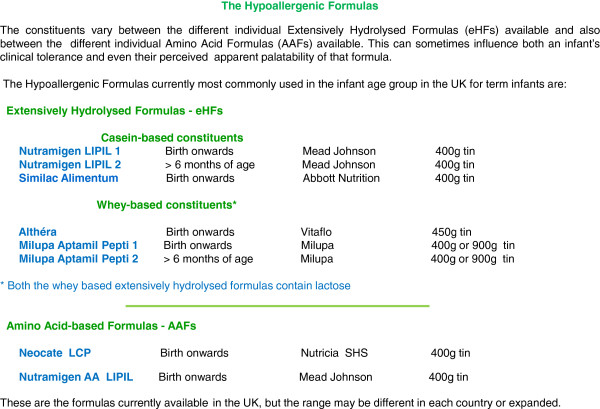
Formulas available for the treatment of CMA in the UK.

Choosing the most appropriate formula (Figure 3, Figure 4; Table 1) for the infant based on the clinical presentation is debated with clear differences between countries. This choice is really a clinical decision which should be based on clinical presentation and the nutritional composition and residual allergenicity of the proposed hypoallergenic formula.

**Table 1:** Guidance on formula choice

**Clinical presentation**	**DRACMA** **1**^**st**^**Choice** **(Fiocchi et al **[2]**)**	**ESPGHAN** **1**^**st**^**Choice** **(Koletzko et al **[7]**)**	**USA** **1**^**st**^** Choice** **(Boyce et al **[10], **Bahtia et al **[22] **American Academy of Pediatrics **[25]**)**	**AUSTRALIAN Consensus** **Panel 1**^**st **^**Choice** **(Allen et al **[11]**)**
Anaphylaxis	AAF	AAF	No recommendation	AAF
Acute urticaria or angioedema	eHF	No specific mention but eHF in general as 1^st^ line treatment for CMA apart from specific indications for AAF	No recommendation	eHF if < 6 months
				Soya if > 6 months
Atopic eczema/dermatitis	eHF	No specific mention but eHF in general as 1^st^ line treatment	No recommendation	eHF if < 6 months
				Soya if > 6 months
				eHF if >6 months if also presenting with faltering growth
Immediate gastrointestinal allergy	eHF	No specific mention but eHF in general as 1^st^ line treatment	No recommendation	eHF if < 6 months
				Soya if > 6 months
				eHF if >6 months if also presenting with faltering growth
Allergic eosinophilic oesophagitis	AAF	AAF (as well as other eosinophilic disorders of the gut)	AAF/hypoallergenic formula (NIAID)	AAF
Gastroesophageal reflux disease (GORD)	eHF	No specific mention but eHF in general as 1^st^ line treatment	No recommendation	eHF if < 6 months
				Soya if > 6 months
				eHF if >6 months if also presenting with faltering growth
Cow’s milk protein-induced enteropathy	eHF	(Severe enteropathy complicated by faltering growth and hypoprotenemia) AAF	eHF/AAF	eHF if < 6 months
				Soya if > 6 months
				eHF if >6 if also presenting with faltering growth
Food protein-induced enterocolitis syndrome (FPIES)	eHF	AAF	Hypoallergenic formula (NIAID) eHF/AAF	eHF
CM protein-induced gastroenteritis and proctocolitis	eHF	No specific mention but eHF in general as 1^st^ line treatment		Gastro-enteritis:
				eHF if < 6 months
				Soya if > 6 months
				eHF if >6 months if also presenting with faltering growth
				Proctitis: eHF
Severe irritability (colic)	eHF	No specific mention but eHF in general as 1^st^ line treatment	Hypoallergenic formula	eHF if < 6 months
				Soya if > 6 months
				eHF if >6 months if also presenting with faltering growth
Constipation/Diarrhoea	eHF	No specific mention but eHF in general as 1^st^ line treatment	No recommendation	eHF if < 6 months
				Soya if > 6 months
				eHF if >6 months if also presenting with faltering growth
Milk-induced chronic pulmonary disease (Heiner’s syndrome)	AAF	No recommendation	No recommendation	No recommendation
Faltering growth	No recommendation	AAF (particularly presenting with enterocolitis complicated by hypoprotenemia and anaemia)	No recommendation	See with other conditions – but defaults to eHF
Breast fed infants – not responding on maternal milk avoidance	No recommendation	AAF	“alternative formulas” eHF/AAF	No recommendation
Multiple food allergies	No recommendation	eHF/AAF	No recommendation	No recommendation
Severe atopic eczema/dermatitis	No recommendation	AAF (particularly if presenting with faltering growth complicated by hypoprotenemia and anaemia)	No recommendation	No recommendation

The problem clinicians face is that it may appear there is a large body of evidence about alternatives to cow’s milk formulae, but most of the research is of low quality and there are a relatively small number of studies about each type of formula. There are very few studies comparing the different formulae in RCTs head to head and the clinical profiles of the patients who improved and did not improve are often very poorly described. This puts the physician and dietitian in a very difficult position when choosing the most appropriate formula for a particular clinical presentation. In some cases choosing a soya or an extensively hydrolysed formula (eHF), which the infant may also react to, may lead to a false negative diagnosis. Alternatively, choosing an amino acid formula (AAF) when not indicated increases the cost burden of managing CMA and may affect development of tolerance (albeit the data is very preliminary at this time) [19,20].

Table 1 summarises the current international guidelines on the use of hypo-allergenic formulae in the diagnosis and management of CMA. It is accepted that the majority of children with CMA will improve on an extensively hydrolysed formula. It is therefore not surprising that in general, the guidelines suggest the use of an AAF, as a first line treatment, only for more severe presentations of CMA such as a history of anaphylaxis, Heiner Syndrome, Eosinophilic Eosophagitis and severe gastro-intestinal and/or skin presentations, usually in association with faltering growth. They recommend the use of an eHF for all other clinical presentations.

Unfortunately, apart from the ESPGHAN guidelines [21], none of the guidelines [2,6,10,11,22] discuss the use of formulae in two important patient groups, namely those with multiple food allergies, and those infants who do not respond to maternal avoidance of cow’s milk (and other suspected allergens) despite a good clinical suspicion that these infants may be reacting to residual allergens. These cases have been reviewed by Hill et al. [23], Niggeman et al. [24] and Van den Plas et al. [8] with data suggesting that these groups may benefit from an AAF. The systematic review by Hill et al. [23] further suggested that those infants presenting with symptoms of CMA whilst exclusively breast fed, who may need a top-up formula or a replacement of breast milk may also benefit from an AAF.

The use of soya formula in the diagnosis and management of CMA is also debated, with clear differences between the Australian consensus panel [11] and the ESPGHAN [7]/AAP [22,25] guidelines. ESPGHAN and AAP acknowledge that only about 10-14% of infants with IgE- mediated CMA will also react to soya, but that this figure is much higher in infants with non-IgE- mediated CMA (25–60%). The two societies therefore recommend that cow’s-milk-based hypoallergenic formulae should ideally be chosen rather than soya formula in the management of CMA. In addition, soya formula contains phytate which may affect nutrient absorption and isoflavonoids in amounts that make soya milk unsuitable for use in all infants under six months of age. Soya can however be used in infants older than 6 months if eHF is not accepted or tolerated, if these hypoallergenic formulae are too expensive, or if there are strong parental preferences (e.g. vegan diet).

In addition, there have been some questions raised regarding the use of hypoallergenic formulae containing lactose in the diagnosis and management of infants and young children with CMA. ESPGHAN [7] advises that adverse reactions to lactose in children with CMA is not reported in the literature and complete avoidance of lactose is not needed in the majority of cases, apart from those children who have an enteropathy with severe diarrhoea where there is a secondary lactose intolerance. Two randomised trials suggested that rice based hydrolysed formula is well tolerated by infants with CMA [26,27] although there are some concerns about the effect of these formulae on weight gain [28].

Therefore, to summarise the above discussion, taking into account the lack of good quality studies in this field:

Breast-feeding is always the preferred way to feed any infant. In any case where there is a need to exclude cow’s milk from the maternal diet and a top-up formula is needed, we suggest in agreement with Hill et al. [23] an amino acid based formula as the B-lactoglobulin levels and peptide sizes of cow’s milk protein in breast milk and those of eHF are similar to the ranges of B-lactoglobulin seen in breast milk [29-33].

AAF is recommended as a first line of treatment for those infants with a history of anaphylaxis to cow’s milk, Heiner Syndrome, Eosinophilic Eosophagitis and severe gastro-intestinal and/or skin presentations, particularly in association with faltering growth.

eHF is recommended as a first line of choice for infants with mild to moderate presentations of CMA e.g. colic, reflux, diarrhoea, vomiting, eczema in the absence of faltering growth. eHF containing whey may not be suitable as a first line of treatment of those infants with possible secondary lactose intolerance [7].

Soya formula can be used in infants over 6 months of age who do not tolerate the eHF, particularly if they are suffering from IgE mediated CMA in the absence of sensitisation to soya.

For the purpose of this paper and management of non-IgE-mediated CMA in UK primary care, Figures 2 and 3 focus on the appropriate choice of hypoallergenic formula to diagnose and manage non-IgE-mediated CMA and an additional three files (Additional file 1: Milk Ladder and Additional file 2: Additional information on milk ladder and Additional file 3: Milk Ladder Recipes) have been uploaded on the cows’ milk challenge and reintroduction procedures to confirm the diagnosis.

#### The role of the dietitian in the diagnosis and management of cow’s milk allergy

The UK NICE guideline [6] highlighted the important role of the dietitian in the diagnosis and management of CMA in terms of assisting with taking the allergy focused diet history, choice of formula, monitoring nutritional status, suggesting nutritional supplements and dietary advice for the breast feeding mother and infant. The particular role of the dietitian in also providing appropriate weaning advice cannot be underestimated. Dietitians can give invaluable advice regarding the level of cow’s milk allergen avoidance that is required i.e. which foods should be omitted and which foods can be tolerated. The role of the dietitian is also to provide written information on suitable substitute foods, recipes, online information, label reading and life-style adjustments [34-36]. Finally, the dietitian plays a central role in organising/designing food challenges to diagnose CMA and determine development op tolerance [34].

#### Determining development of tolerance to cow’s milk

There is no ideal time for testing for development of tolerance, but it is generally accepted that infants with a proven cow’s milk allergy diagnosis should remain on a cow’s milk protein free diet until 9–12 months of age and for at least 6 months [10] prior to reintroduction of cow’s milk into their diets.

Hospital challenge or onward referral to secondary/tertiary care should be considered if either of the following is true:

1) there has ever been suspicion of an acute onset of symptoms following ingestion,

2) current atopic eczema and positive specific serum IgE for CMP.

See Figure 3.

The NICE UK Food Allergy Guideline states that no child with IgE-mediated food allergy should have a food challenge in primary care or community settings. All those remaining children diagnosed as mild-moderate non-IgE-mediated CMA are suitable for a home challenge. Guidance on how to perform this challenge/reintroduction is given in the additional three uploaded files.

##### For the purpose of this paper we have used the following terminology

Food challenge is the term used for deliberate milk exposure for the purposes of an initial diagnosis of CMA (usually after a 2–4 week period of avoidance) [8] and can be done at home or in hospital, depending on the circumstances.

Food challenge is also the term used for deliberate milk exposure for testing for resolution of CMA after an extended period of avoidance. For IgE mediated allergy, this is usually done in hospital as a baked milk challenge or a standard milk challenge, but can be done at home in certain circumstances.

Food reintroduction is the term used for the gradual reintroduction of cow’ milk after an extended period of avoidance. This is usually done at home, in non IgE mediated allergy, as a “milk ladder”.

##### Challenges/Reintroduction

The home reintroduction performed in children with non-IgE-mediated cow’s milk allergy could also be referred to as the Milk Ladder.

The Milk Ladder is based around the knowledge that Cow’s Milk Proteins (CMPs) include casein protein fractions (*αs1-,αs2,β-*&*κ-caseins*) and whey protein fractions (*α-lacto globulin* &*β-lacto globulin*) which contain sequential and conformational epitopes. Thermal processing provokes changes to the proteins’ conformational structures (mainly whey proteins) and may lead to a change in allergenicity of CM containing foods. Furthermore, the interaction between food proteins and other components such as carbohydrates and fats during heating of a complex food (food matrix effects) may reduce the milk protein allergenicity [37-39].

Several studies have observed that children with transient IgE-mediated CMA produce milk specific IgE antibodies against conformational IgE-binding epitopes that are destroyed during extensive heating or food processing [38,40]. Clinical trials have reported that almost 75% of children with IgE-mediated CMA may tolerate baked milk containing foods like muffins, cakes, breads and waffles [38]. In addition the inclusion of these foods in these children’s diets seems to accelerate development of tolerance to CMA compared to the complete milk exclusion approach [37]. The role of processing the development of tolerance in children with non-IgE-mediated CMA has not been investigated, but it does seem to be a safe approach to perform food challenge in primary care. There is really very little evidence for devising milk ladders in terms of the effect of heating and dose on the allergenicity of specific foods, the next best option is therefore to devise guidelines on the basis of clinical/expert opinion.

A group of dietitians from the UK Wessex Allergy Network have worked with industry to devise a milk ladder in terms of allergenicity of foods, taking into account the type of milk used in the recipes (whey powder vs. milk), the temperature of heating and the time of heating. Affected individuals and their families should, however, be advised that the classification of milk-containing foods from lowest to greatest allergenicity in a ‘milk ladder’ is not perfect, but based on the best available information.

The authors suggest that the milk ladder is used in the following way:

Most children/infants will start at step one of the ladder. However, some children/infants might have consumed some of the foods on the “ladder” already and would therefore not need to start at step one of the ladder e.g. a child who is already consuming baked milk containing muffins (step 3) could start with the introduction of pancakes (step 4).

In some cases the dietitian/clinician may prefer to start the ladder with smaller quantities than suggested e.g. a crumb of a malted milk biscuit, ¼ malted milk biscuit or ½ malted milk biscuit.

If the food in a certain “step” of the ladder is tolerated, the authors advise to continue eating the food and try the food suggested in the next step.

Each step of the ladder can be conducted over any length of time (for example one day or one week) as indicated by the dietitian/physician. The duration of each step will be based on the characteristics of each individual case.

For each “step” in the ladder the milk ladder development group have provided a commercially available option and a home-made option. This gives mothers the option to choose what they would like to give to their children and to adjust the texture to the developmental milestones of the child/infant (e.g. the pasta dishes can be mashed up for younger children).The ladder can also alternate between commercial and home-made options e.g. buy the biscuits but bake the muffins.

There are different levels of care/support in the UK and one of the reasons for producing this guidance is to make the dietary aspects of dealing with CMA more uniform and provide practical guidance in areas where there is a lack of dietetic support.

If symptoms recur it is suggested that the challenge is repeated at 4–6 monthly intervals. If milk is tolerated up to a certain threshold either by amount or degree of heating / cooking (which reduces the allergenicity), the level at which it is tolerated should be continued and a challenge with larger amounts or less heated/cooked undertaken again in 4–6 months. It is important to understand that tolerance to milk containing foods relates only to foods tolerated on the ladder. E.g. if a child reacts to milk chocolate, then consumption of biscuits, cakes, pancakes, baked milk dishes and pizza should be safe and should be eaten regularly in the diet. Foods such as chocolate, yoghurt, cheese and milk should be avoided in this particular case [35].

### Testing of the algorithms

The initial sets of guidelines were trialed in the Northern Ireland Region of the UK NHS with feedback. The authors also involved an MSc student from the University of Southampton who conducted a qualitative study with primary and secondary care clinicians (GPs, dietitians and paediatricians – publication in preparation). The Chair of the Primary Care Group of the British Society of Allergy and Clinical Immunology and a European Secondary Care Clinician were asked to review the algorithms. The authors have taken on board the comments of all those involved and adjusted the initial guidelines to what is shown in this publication.

## Conclusion

Cow’s milk allergy can present with a spectrum of acute or delayed symptoms that can be mild to moderate or severe in nature. Symptoms may affect the respiratory, cutaneous and gastrointestinal systems, or a combination of these systems. Diagnosis and management of non-IgE-mediated CMA can take place in primary care; however all infants on a cow’s milk exclusion diet should ideally be referred to a dietitian, preferably before weaning onto solid food takes place. Referral to secondary care should be made as per the proposed algorithms, adapted from the UK NICE Food Allergy guideline.

## Competing interests

Carina Venter has received funding for educational lectures and research grants from Mead Johnson, Danone, Vitaflo, Pfizer and GlaxoSmithKline. Trevor Brown has received honoraria for giving education lectures for Mead Johnson and Danone. Joanne Walsh received funding for consultancy work and educational lecture fees from Mead Johnson, Danone and Thermo Fisher. Neil Shah no competing interests Adam T Fox has received funding for consultancy work, lecture fees and research grants from Mead Johnson and Danone.

## Author’s contributions

CV was actively involved in developing the algorithms; she led the development of the Milk Ladder by the Wessex Allergy Network Group and prepared the manuscript. TB initially set up this guideline development group and subsequently has continued to contribute to the on-going development of this UK primary care-focused CMA guideline and in the preparation of this manuscript. JW has helped in development of the algorithms and preparation of the manuscript. NS helped in preparation of the manuscript and advised on the non-IgE components of this publication. ATF helped in development of the algorithms and preparation of the manuscript. All authors read and approved the final manuscript.

## Supplementary Material

Additional file 1 Home challenge or hospital challenge to confirm the diagnosis of cow’s milk allergy. Click here for file

Additional file 2 Milk ladder additional information. Click here for file

Additional file 3 Recipes to go with milk ladder. Click here for file
