# Supplementary material for: Diagnosis and management of non-IgE-mediated cow’s milk allergy in infancy - a UK primary care practical guide
Source: Clin Transl Allergy. 2013 Jul 8;3:23. doi: 10.1186/2045-7022-3-23 (PMC3716921; doi:10.1186/2045-7022-3-23)
Supplement: Additional file 1 — Home challenge or hospital challenge to confirm the diagnosis of cow’s milk allergy. [file 2045-7022-3-23-S1.docx]

| **Clinical presentation**  Table 1: Guidance on formula choice | **DRACMA**  **1^st^ Choice**  **(Fiocchi et al. ^2^)** | **ESPGHAN**  **1^st^ Choice (Koletzko et al. ^7^)** | **USA**  **1^st^ Choice**  **(Boyce et al.^10^ and Bahtia et al^21^)** | **AUSTRALIAN Consensus Panel 1^st^ Choice**  **(Allen et al.^11^)** |
| --- | --- | --- | --- | --- |
| Anaphylaxis^1^ | AAF | AAF | No recommendation | AAF |
| Acute urticaria or angioedema^1^ | eHF | No specific mention but eHF in general as 1^st^ line treatment for CMA apart from specific indications for AAF | No recommendation | eHF if < 6 months  Soya if > 6 months |
| Atopic eczema/dermatitis^1^ | eHF | No specific mention but eHF in general as 1^st^ line treatment | No recommendation | eHF if < 6 months  Soya if > 6 months  eHF if >6 months - and also faltering growth |
| Immediate gastrointestinal allergy^1^ | eHF | No specific mention but eHF in general as 1^st^ line treatment | No recommendation | eHF if < 6 months  Soya if > 6 months  eHF if >6 months – and also faltering growth |
| Allergic eosinophilic oesophagitis^1^ | AAF | AAF (as well as other eosinophilic disorders of the gut) | AAF/hypoallergenic formula (NIAID) | AAF |
| Gastroesophageal reflux disease (GORD) ^1^ | eHF | No specific mention but eHF in general as 1^st^ line treatment | No recommendation | eHF if < 6 months  Soya if > 6 months  eHF if >6 months – and also faltering growth |
| Cow’s milk protein-induced enteropathy^1^ | eHF | (Severe enteropathy complicated by faltering growth and hypoprotenemia) AAF | ehF/AA (2008) | eHF if < 6 months  Soya if > 6 months  eHF if >6 months- and also faltering growth |
| Food protein-induced enterocolitis syndrome (FPIES) ^1^ | eHF | AAF | Hypoallergenic formula (NIAID)  eHF/AA (2008) | eHF |
| CM protein-induced gastroenteritis and proctocolitis^1^ | eHF | No specific mention but eHF in general as 1^st^ line treatment |  | Gastro-enteritis:  eHF if < 6 months  Soya if > 6 months  eHF if >6 months – and also faltering growth  Proctitis: eHF |
| Severe irritability (colic) ^1^ | eHF | No specific mention but eHF in general as 1^st^ line treatment | Hypoallergenic formula (2000) | eHF if < 6 months  Soya if > 6 months  eHF if >6 months – and also faltering growth |
| Constipation/Diarrhoea^1^ | eHF | No specific mention but eHF in general as 1^st^ line treatment | No recommendation | eHF if < 6 months  Soya if > 6 months  eHF if >6 months – and also faltering growth |
| Milk-induced chronic pulmonary  disease (Heiner’s syndrome) ^1^ | AAF | No specific mention but eHF in general as 1^st^ line treatment | No recommendation | No recommendation |
| Faltering growth | No | AAF  ^(^particularly presenting as enterocolitis complicated by hypoprotenemia and anaemia) | No recommendation | See with other conditions – but defaults to eHF |
| Breast fed infants – not responding on maternal milk avoidance^2,3,4^ | No recommendation |  | “alternative formulas” eHF/AA (2000) | No recommendation |
| Multiple food allergies^2,3,4^ | No | eHF/AAF | No recommendation | No recommendation |
| Severe atopic eczema/dermatitis^2,3,4^ | No recommendation | AAF ( particularly if faltering growth complicated by hypoprotenemia and anaemia) | No recommendation | No recommendation |
