# Supplementary material for: Diagnosis and management of non-IgE-mediated cow’s milk allergy in infancy - a UK primary care practical guide
Source: Clin Transl Allergy. 2013 Jul 8;3:23. doi: 10.1186/2045-7022-3-23 (PMC3716921; doi:10.1186/2045-7022-3-23)
Supplement: Additional file 2 — Milk ladder additional information. [file 2045-7022-3-23-S2.docx]

| Food  **Additional file 2: Milk ladder additional information** | Amount | Milk protein content | Source of milk protein | Temp | Time |
| --- | --- | --- | --- | --- | --- |
| **Malted milk biscuits** | 1 Malted milk biscuit  2 Malted milk biscuits | Commercial products: Average 0.024 g (24 mg) range: 0.02 – 0.03g (20 mg – 30mg ) per biscuit  *Home-made recipe: ¼ biscuit: 0.023 g (23 mg)*  *Milk equivalent: 0.72 mls*  Average: 0.048 g (48 mg)  *Home-made recipe: ½ biscuit: 0.046 g (46 mg)*  *Milk equivalent: 1.44 mls* | Milk powder/whey powder | 180° C | 6.5 min |
| **Garibaldi biscuits/Digestives** *(do check that they contain MILK)* | ½ biscuit  1 biscuit | Commercial products: Average: 0.111 g (110.5 mg) range: 0.102 – 0.119 g (102 – 119 mg)  *Home-made recipe: 1 biscuit: 0.095 g (95 mg)*  *Milk equivalent: 2.9 mls*  Average: 0.222 g (222 mg)  *Home-made recipe: 2 biscuits: 0.19 g (190 mg)*  *Milk equivalent: 5.8 mls* | Whey powder  Lactose and skimmed milk powder | 180° C | 5 min |
|  |  | *In some cases, it may be advisable to start with 1/4 or 1/3 muffin in order to reduce the sharp increase from 5.8 mls in 2 biscuits – 12.5 mls milk in half a muffin.* |  |  |  |
| **Mini muffins/cup cakes**  *Note that Scotch Pancakes contain LESS milk than muffins but baked for a much shorter time* | ½ muffin /cup cake (30g)  1 Muffin (30 g) | Commercial products: Average: 0.415 g (415 mg)  range: 0.28 g – 0.55 g (280 mg – 550 mg)  *Home made recipe: ½ muffin: 0.413 g (413 mg)*  *Milk equivalent: 12.5 mls*  Commercial products: Average: 0.830 (830 mg)  *Home made recipe: 1 muffin: 0.825 g (825 mg)*  *Milk equivalent: 25 mls* | Whole milk/milk powder | 180 – 200° C | 20 – 25 min |
|  |  | IN THE NEXT STEP THE AMOUNT OF MILK IS REDUCED AS THE TIME OF HEATING AND THE TEMPERATURE IS REDUCED |  |  |  |
| **Scotch pancakes –** *it is recommended to look out for scotch pancakes containing milk protein rather than whey powder* | 1 Scotch Pancake  3 Scotch pancakes | Commercial products: 0.141 g (141 mg) milk protein or 1.0- 1.8 g (1000 – 1800 mg) whey protein  *Home-made: ½ scotch pancake: 0.132 g (132 mg milk protein)*  *Milk equivalent: 4 mls*  Commercial products: 0.432 g (432 mg) of milk protein or about 3-4.5 g whey powder  *Home made recipe: 2 Scotch pancakes: 0.53 g (530 mg protein)*  *Milk equivalent: 16 mls* | Milk protein or Whey or  Skimmed milk | > 72° C | 20 sec – 5 min |
|  |  | *In certain cases, it may be advisable to start with ½ child’s portion of shepherd’s pie followed by a full portion.* |  |  |  |
| **Shepherds Pie** | Shepherds Pie | Commercial products: Average milk protein/200 g portion 2.121 g (2121 mg) range: 1.197 g – 3.185 g (1197 mg – 3185 mg)  *Home-made recipe: 1 Child’s portion: 1.9 g (1900 mg) milk protein*  *Milk equivalent: 18.8 ml and 5 g cheese* | Milk, Cream, Butter | 180 – 200° C | 30 min |
| **Lasagne** | Lasagne | Commercial products: Average milk protein/200 g portion 2.45 g (2450 mg) range: 0.022 g – 2.53 g (220 mg – 2530 mg)  *Home-made recipe: 1 Portion 2.6 g milk protein*  *Milk equivalent per child’s portion: 41.3ml + 5 g cheese* | Milk, Cream, Butter, Cheese. | 180 – 200° C | 40 min |
| **Pizza** – *we recommend that a pizza is chosen that does not contain milk in the base* | ½ Mini Pizza  1 Mini Pizza | Commercial products: 1.75 g  *½ Home-made pizza: 1.875 g (1875mg) milk protein*  *Milk equivalent: 7.5 g cheese*  Commercial products: 3.5 g per mini pizza  *1 Home-made pizza: 3.75 g (3750mg) milk protein*  *Milk equivalent: 15 g cheese* | Milk protein derived from milk and cheese | 200° C | 8- 10 min |
| Milk chocolate | 10 g Chocolate  Milk choc buttons (1/2 bag or 35 g) | Commercial products: 0.6 milk protein (600 mg) per 10g  Milk equivalent per portion: 18.2 mls  Commercial products: 2.1 g milk protein (2100 mg) per 35 g  Milk equivalent per portion: 63.7 mls | Pasteurised milk | NA | NA |
| Yogurt | 1 pot yoghurt (125 ml) | Commercial products: 2.6-4.7g milk protein (2600-4700 mg) per pot  Milk equivalent per portion: 78.7 mls – 142.9 mls | Pasteurised milk | 98° C | 2 min 30 sec |
| Cheese – *most hard cheese contains about 20% - 25% protein and can be used at this point* | 25 g cheese | Commercial products: 4.4g milk protein (4400 mg) in one babybel  Milk equivalent per portion: 25 g cheese | Pasteurised milk | 72° C | 15 sec |
| Sterilised milk | 100 mls  200 mls | 3.3 g milk protein - 3.6 g milk protein (3300-3600 mg)  6.6g milk protein – 7.2 g milk protein (6600-7200 mg) | Sterilised milk  Infant formula (tetra packs) | pre-heated to 50^o^C, then homogenised  110-130ºC  138 – 140°C | 10 – 30 min |
| Pasteurised milk | 100 mls  200 mls | 3.3 g milk protein - 3.6 g milk protein (3300-3600 mg)  6.6g milk protein – 7.2 g milk protein (6600-7200 mg) | Pasteurised milk  Infant formula (powder) | 57 – 68° C | 15-20 seconds |

This milk ladder was produced based on information of commercial products in the UK. The information was correct at the time of submission for publication (April 2013).
