# Supplementary material for: Diagnosis and management of non-IgE-mediated cow’s milk allergy in infancy - a UK primary care practical guide
Source: Clin Transl Allergy. 2013 Jul 8;3:23. doi: 10.1186/2045-7022-3-23 (PMC3716921; doi:10.1186/2045-7022-3-23)
Supplement: Additional file 3 — Recipes to go with milk ladder. [file 2045-7022-3-23-S3.docx]

**Additional file 3: Recipes to go with milk ladder**

**Biscuit (“malted milk”)**

Makes 40 small biscuits

**Ingredients**

110g unsalted butter - softened

110g caster sugar

175g self-raising flour

50mls evaporated milk

Water if mixture is too dry

**Method**

Preheat oven to 180C (160C for fan ovens)

In a large bowl, cream the butter and sugar and evaporated milk until pale

Mix in sifted flour

This will make a stiff dough - if too dry add a small amount of water

Divide dough into 2 and then into 2 again

Out of each quarter of the dough - make 10 small equal size balls

Place on baking tray covered with greased baking paper

Cook for 15 minutes or until golden at the edges but still a little soft

Transfer to cooling rack

Dough not used can be wrapped in cling film and frozen

Cooked biscuits can also be frozen

**Variations**

Add any of the following - dairy free chocolate drops, grated lemon and/or orange zest and a small amount of juice, any spices such as cinnamon, dried fruit such as raisins, apricots, dates

**Nutrition Information**

Milk equivalent per biscuit: 2.88 ml (1/4 biscuit = 0.72 mls and ½ biscuit = 1.44 mls)

Milk protein per biscuit : 0.095g

**Gluten and wheat free alternative:**

For a gluten and wheat free biscuit replace the wheat flour with 175g gluten and wheat free self-raising flour

**Mini muffins/cupcakes**

Makes 8 muffins

THESE MUFFINS/CUPCAKES can also be used for a BAKED MILK Challenge

In order to provide 50 ml of milk (as per Anna Nowak-Wegzryn studies*) 2 muffins need to be eaten.

*Nowak-Wegrzyn A, Assa'ad AH, Bahna SL, Bock SA, Sicherer SH, Teuber SS. Work Group report: oral food challenge testing. J Allergy Clin Immunol 2009 Jun;123(6 Suppl):S365-S383

**Ingredients**

132g Wheat or Wheat free flour

40g Cocoa powder

1½ tsp Baking powder

¾ tsp Bicarbonate of soda

100g Soft brown sugar

40ml Any oil

70 ml Prune juice/Apple Puree

200ml Milk

½ tsp Vanilla extract

**Method**

Mix all the dry ingredients together

Mix the oil, apple puree/prune juice, milk and vanilla extract together

Add the fluid to the dry ingredients and mix lightly.

Scoop mix into muffin pans.

Bake at 180 C for 20 min.

**Variations**

Can be served with icing made with a suitable dairy free margarine or dairy free cream cheese.

**Nutrition Information**

Milk per muffin: 25mls (1/2 muffin = 12.5 mls)

Milk protein per muffin: 0.825 g

**Scotch pancakes:**

Recipe provides 18 pancakes

**Ingredients**

120g self-raising flour
small pinch salt
30g caster sugar
1 egg
144 ml milk

**Methods**

1. First grease your pan - use oil as butter will burn, and then heat.
2. Sift the flour into a bowl, add the pinch of salt and the sugar.
3. Crack the egg into the milk, and whisk.
4. Pour the egg and milk liquid into the dry ingredients, and mix to form a smooth batter.
5. Test that the pan is hot enough by putting a teaspoon of batter onto it.

6. Use a tablespoon to drop the batter onto the pan.

7. When the surface of the batter has become covered in bubbles get ready to flip them over using a spatula.
8. When cooked remove the pancakes from the pan and wrap in a clean tea towel to keep moist.

**Egg free or wheat free alternative**

For an egg free alternative replace the egg with ½ large banana or use the instructions on an egg replacer.

For a wheat free pancake replace the wheat flour with wheat free flour.

**Nutrition Information**

Milk per pancake: 8ml (1/2 scotch pancake = 4 mls)

Milk protein per pancake: 0.26 g

**Shepherd’s pie Recipe**

**Excludes:** Egg, Wheat, Gluten, Soya, Nuts **Serves:** 8 children

**Ingredients**

- 650g [lamb mince or beef mince](http://www.bbc.co.uk/food/lamb_mince)
- 1 tbsp [vegetable oil](http://www.bbc.co.uk/food/vegetable_oil)
- 1 large [onion](http://www.bbc.co.uk/food/onion), finely chopped
- 600ml [vegetable stock](http://www.bbc.co.uk/food/vegetable_stock)
- ½ tsp mixed [herbs](http://www.bbc.co.uk/food/herb)
- 1 tbsp [tomato purée](http://www.bbc.co.uk/food/tomato_puree)
- sea salt and freshly ground [black pepper](http://www.bbc.co.uk/food/black_pepper)
- 900g [potatoes](http://www.bbc.co.uk/food/potato), peeled and chopped
- 55g [butter](http://www.bbc.co.uk/food/butter)
- 100mls [milk](http://www.bbc.co.uk/food/milk)
- 5 g skimmed milk powder (dissolved into milk)
- 40 g grated cheddar cheese

**Nutrition information**

Milk equivalent per child’s portion: 18.8 ml milk and 5 g cheese

Milk protein per child’s portion: 1.9 g

**Lasagne**

**Excludes: E**gg, Nuts, Soya **Contains:** Wheat, Gluten, Dairy **Serves:** 8 children

**Ingredients**

[oil](http://www.bbc.co.uk/food/oil), to fry

450g lean [beef mince](http://www.bbc.co.uk/food/beef_mince)

1 [onion](http://www.bbc.co.uk/food/onion), finely chopped

1 red [pepper](http://www.bbc.co.uk/food/pepper), seeds removed, finely chopped

1 green [pepper](http://www.bbc.co.uk/food/pepper), seeds removed, finely chopped

150g [mushrooms](http://www.bbc.co.uk/food/mushroom), sliced

200g [tomato purée](http://www.bbc.co.uk/food/tomato_puree)

2 bay leaves

2 x 400g tin [chopped tomatoes](http://www.bbc.co.uk/food/chopped_tomatoes)

1 tsp dried thyme, parsley and [oregano](http://www.bbc.co.uk/food/oregano)

salt and freshly ground [pepper](http://www.bbc.co.uk/food/pepper)

350g lasagne sheets

**Nutrition information**

Milk per child’s portion: 41.3 ml + 5 g cheese

Contains cow’s milk protein – 2.6 g per child’s serving

**Gluten and wheat free alternative:**

For a gluten and wheat free lasagne replace the lasagne sheets with 300g dried wheat free [pasta](http://www.bbc.co.uk/food/pasta) sheets

**Mini pizza**

**Ingredients and method**

Use bread or pitta bread

Tomato purée to cover the bread or pitta bread

Add a variety of vegetables as preferred

Cover the pizza with 15g (1/2 oz) of cheese (mozzarella or cheddar or a mix)

Bake for 5-10 min at 200 C.

**Wheat free alternative**

For a wheat free pizza, use a wheat free pitta bread or a corn tortilla.

**Nutrition Information**

Milk protein content per “pizza” = 3.75 g milk protein

Milk equivalent per portion: 15 g cheese
